# Pharmacological and psychotherapeutic interventions for management of poststroke depression: A Bayesian network meta-analysis of randomized controlled trials

**DOI:** 10.1097/MD.0000000000006100

**Published:** 2017-02-17

**Authors:** Xuejun Sun, Linghui Deng, Shi Qiu, Xiang Tu, Deren Wang, Ming Liu

**Affiliations:** a Second Department of Psychiatry, Kangning Hospital, Anshan, Liaoning; b Stroke Clinical Research Unit, Department of Neurology; c Department of Urology, Institute of Urology, West China Hospital, Sichuan University, Chengdu, Sichuan, China.

**Keywords:** Bayesian network meta-analysis, Hamilton depression scale, pharmacological, poststroke depression, psychotherapeutic

## Abstract

**Introduction:**

Poststroke depression (PSD) constitutes an important complication of stroke, leading to great disability as well as increased mortality. Since which treatment for PSD should be preferred are still matters of controversy, we are aiming to compare and rank these pharmacological and nonpharmacological interventions.

**Methods and analysis:**

We will employ a network meta-analysis to incorporate both direct and indirect evidence from relevant trials. We will search PubMed, the Cochrane Library Central Register of Controlled Trials, Embase, and the reference lists of relevant articles for randomized controlled trials (RCT) of different PSD treatment strategies. The characteristics of each RCT will be summarized, including the study characteristics, the participant characteristics, the outcome measurements, and adverse events. The risk of bias will be assessed by means of the Cochrane Collaboration's risk of bias tool. The primary outcome was change in Hamilton Depression Scale (HAMD) score. Secondary outcomes involve patient response rate (defined as at least a 50% score reduction on HAMD), and remission rate (defined as no longer meeting baseline criteria for depression). Moreover, we will assess the acceptability of treatments according to treatment discontinuation. We will perform pairwise meta-analyses by random effects model and network meta-analysis by Bayesian random effects model.

**Conclusion:**

Formal ethical approval is not required as primary data will not be collected. Our results will help to reduce the uncertainty about the effectiveness and safety of PSD management, which will encourage further research for other therapeutic options. The review will be disseminated in peer-reviewed publications and conference presentations.

**PROSPERO registration number:**

CRD42016049049

## Abstract 2

Supplemental Digital Content is available in the text

**Key Points**

1. This systematic review and network meta-analysis will evaluate the effectiveness and safety of pharmacological and nonpharmacological treatment for poststroke depression.
2. The protocol has been created according to the published PRISMA-P guidelines. This review will be based on a comprehensive search strategy and the outcomes will provide clinicians, patients, and caregivers with tailored evidence to inform their decision-making.
3. A potential difficulty in the conduct of our study is that some extent of clinical heterogeneity considering patient characteristics exist. Furthermore, the ability to explore heterogeneity may be limited in case of the small number of included studies.

## 1 Introduction

Stroke is one of the top causes of death and disability globally, and depression is a common sequelae of stroke. Poststroke depression (PSD) occurs in 31% stroke survivals according to a recent meta-analysis,^[1]^ giving rise to a great burden to patients as well as their families. Several studies suggested PSD was associated with reduced quality of life and increased mortality.^[2–5]^

The diagnosis of PSD can be complicated, because of the overlap of some physical symptoms. Stroke survivals with cognitive and language impairments can be more troublesome. Moreover, various screening tools and diagnostic standard also contribute to the challenge of identification of PSD. Thus, although PSD has detrimental impacts on rehabilitation, only a small amount of patients got properly diagnosed and receive relevant treatment.^[5,6]^

PSD is unique because stroke, depression, and the resultant disability often occur abruptly, thus the relationship between stroke and following depression can be quite complicated accordingly. The pathogenesis of PSD remains controversy about whether PSD is a direct consequence of neuroanatomical impairment, or indirectly due to the patients’ abnormal psychological response to a life-threatening cerebrovascular accident.^[7]^ Many factors like stroke severity, lesion locations, functional, and cognitive impairment may contribute to the development of PSD.^[8]^ Studies demonstrated that the incidence of depression was significantly higher in stroke survivals compared with reference population without stroke,^[2]^ even with comparable physical impairments.^[9]^ Moreover, PSD was more likely characterized by sad facial expression and vegetative symptoms compared with other kinds of depression.^[10]^ In return, evidence suggests that depression severity was an independent predictive factor of severity of impairment in daily activities among stroke survivals.^[11]^ Given that PSD differs in potential unique ways, it may be inappropriate to simply extrapolate data of general depression population to PSD patient management.

Several therapeutic strategies for PSD were proved to be effective, including pharmacological and nonpharmacological approaches (eg, psychotherapy, electroconvulsive therapy [ECT]). Antidepressants are most studied strategies and the best studied agents are citalopram, nortriptyline, fluoxetine, and sertraline.^[12]^ Major goals of PSD treatments include reducing depressive symptoms and getting complete remission (no longer meeting baseline criteria for depression).^[13]^ Meta-analysis found antidepressants to be significantly effective in reducing depressive symptoms.^[13,14]^ However, there is no clear evidence to recommend antidepressants in terms of getting complete remission of depression when assessed by Diagnostic and Statistical Manual of Mental Disorders (DSM) or Hamilton Depression Rating Scale (HAMD).^[13,15]^ Although selective serotonin reuptake inhibitors (SSRIs) are gaining popular as 1st-line treatment for PSD and late-life depression,^[12]^ but no study provides conclusive evidence on the superiority of SSRIs over any other treatments, nor strong data recommending 1 particular SSRI over another for PSD management.

Despite the numerous therapeutic interventions including both pharmacological and nonpharmacological approaches evaluated in previous randomized controlled trials (RCT) to treat PSD, the majority has not been quantitative analyzed in head-to-head comparisons. Thus, we employed a network meta-analysis (NMA) of all RCTs of treatment approaches for PSD, including pharmacological, nonpharmacological, and combine of those, to perform a comprehensive ranking of all available treatments for PSD.

## 2 Method

This protocol was prepared underlying the Preferred Reporting Items for Systematic Review and Meta-Analysis Protocols (PRISMA-P) guidance.^[16]^ Our report will be in line with the recommendations of the PRISMA Extension Statement for Reporting of Systematic Reviews Incorporating Network Meta-analyses of Health Care Interventions.^[17]^ The NMA protocol was registered with the international prospective register of systematic reviews (PROSPERO; Registration number CRD42016049049)

### 2.1 Eligibility criteria

#### 2.1.1 Types of studies

We will only involve RCTs and quasi-RCTs using the HAMD for assessing the depression degree of patients, with data of score change between pre- and posttreatment, or response or remission rate to the treatment. Studies should be available in full-text and peer-reviewed.

#### 2.1.2 Types of participants

Participants need to own the following characteristics: adults 18 years or older; a clinical diagnosis of stroke, ischemic, or hemorrhagic; and a clinical diagnosis of PSD, by specific criteria (eg, DSM-III, DSM-III-R, and DSM-IV) or depression scales (eg, HAMD). The criteria have been changed over time, thus we will record how the authors define PSD severity for each trial.

### 2.2 Interventions

Interventions are pharmaceutical agents (alone or in combination with other agents), psychological therapy, electroconvulsive therapy, active repetitive transcranial magnetic stimulation, acupuncture therapy, social support, or the combined therapy of any above. Specific pharmacological agents include antidepressants, traditional Chinese medicine. We will analysis antidepressants according to their substance class (eg, fluoxetine belongs to SSRIs), and categorized pharmacological interventions into these following groups: SSRIs, tricyclic and tetracyclic antidepressants, noradrenaline reuptake inhibitor, serotonin–noradrenaline reuptake inhibitor, monoamino oxidase inhibitors, and traditional Chinese medicines. The content of the psychotherapy could vary from simple counseling to specific programs helping patients improving their problem-solving skills and adjusting to the emotional influence on stroke in daily life.

### 2.3 Outcome measures

Primary outcomes: The mean change in HAMD scale data from baseline to endpoint is going to be considered as our primary analysis. For trials including multiple outcome timepoint, we will give priority to the timepoint of treatment duration used in the individual original trial as endpoint of the study (eg, the treatment duration was 9 weeks while follow-up lasted for 2 years). An approximation of the mean will be used to evaluate the outcomes, if data are merely available in graphic format. The highest standard deviations in the HAMD scores from the other trials will be recruited when data are presented without standard deviations.

Secondary outcomes: secondary outcomes will involve patient response rate (defined as at least a 50% score reduction on HAMD), and remission rate (defined as no longer meeting baseline criteria for depression). Moreover, we will assess the acceptability of treatments according to treatment discontinuation, defined as the proportion of participants who leave the trial early for any reason, and the treatment tolerability, defined as the proportion of participants who leave the study early due to adverse events.

### 2.4 Search strategy

Searches for published RCTs will be undertaken, compiled from the underlying databases: PubMed (MEDLINE), EMBASE, and the Cochrane Library Central Register of Controlled Trials (CENTRAL). We will execute free-text terms with various synonyms and a combination of controlled terms (Medical Subject Heading), and the search strategy for each database is detailed in online supplementary appendix. We will only identify RCTs published in English and up to November 1st, 2016. Additionally, we will manually check relevant reviews in the discipline as well as the reference lists of retrieved publications. We are intending to obtain additional gray literature from personal communication from experts in the field, reviewing the reference lists of correlated articles, conference proceedings, and looking for results of unpublished trials. We will contact authors of unpublished work and authors of published trials in order to clarify information when necessary.

### 2.5 Study selection

**Figure 1:**
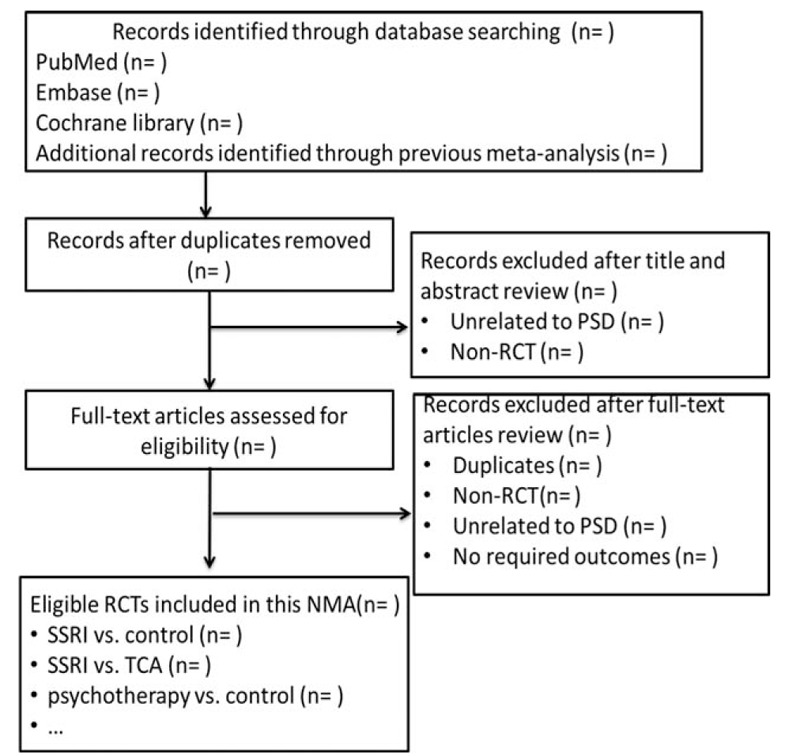
PRISMA flow diagram. NMA = network meta-analysis, PRISMA = Preferred Reporting Items for Systematic Review and Meta-Analysis, PSD = poststroke depression, RCT = randomized controlled trial, SSRI = selective serotonin reuptake inhibitor, TCA = tricyclic antidepressant.

The title and abstract will be initially identified by 2 independent reviewers (XJS and LHD) for potentially eligible articles. The full text for each article which appears to meet the inclusion criteria will be obtained after checking all titles and abstracts. Duplicate studies will be removed after full-text screening and reference checking. Multiple reports of the same work will be resolved by involving the most recently published article. Both reviewers will meet and review their selections after completion. Discrepancies will be handled by consensus. If consensus cannot be reached, a 3rd designated reviewer will provide a recommendation (ML). Figure 1 shows the proposed structure for the flow diagram.

### 2.6 Data extraction

Four raters (XJS, LHD, SQ, and XT) will extract the relevant information from the included studies with a pretested data extraction form from the eligible studies. Data items to be extracted include: study characteristic (location, setting, center, sample size, intervention, follow-up period, drop-out rate, and population); patient characteristic (age, gender, baseline HAMD score, hemisphere stroke status, depression class, and time since stroke); adverse event (death, central nervous system events, gastrointestinal events, psychiatric events, and vascular events); and primary and secondary outcome measures (Tables 1 and 2). In case of disagreement in evaluating the methodological quality of the study, we will try to handle it by consensus. If consensus cannot be reached, a 3rd designated reviewer (ML) will be invited to arbitrate.

**Table 1:**
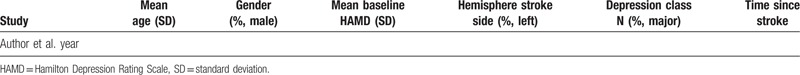
Summary of patient characteristics.

**Table 2:**
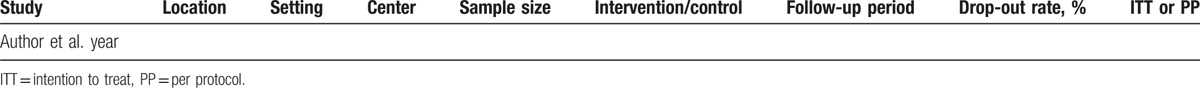
Summary of study characteristics.

### 2.7 Risk of bias and quality appraisal

The validity of the NMA is going to elucidate by qualitative appraisal of study designs and methods. We will consider the methodological quality of the RCTs by means of the Cochrane Collaboration's risk of bias tool.^[18]^ The tool is based on the underlying 8 potential sources of bias: random sequence generation; allocation concealment; blinding of the participants; blinding of the outcome assessors; incomplete outcome data; missing data; selective outcome reporting; and other bias. All 8 domains will be assessed and demonstrated in Table 3 for each study. If we recruit at least 10 studies, a funnel plot for each intervention outcome will be constructed to evaluate the potential publication bias.^[19]^ We will perform visual inspection as well as Begg test^[20]^ and Egger test^[21]^ to determine the funnel asymmetry. The GRADE will be carried out to evaluate the evidence quality of estimates derived from NMA. Direct evidence from RCTs starts at high quality and can be downgraded based on risk of bias, imprecision, indirectness, inconsistency (or heterogeneity), and publication bias to levels of moderate, low, and very low quality.^[22]^

**Table 3:**
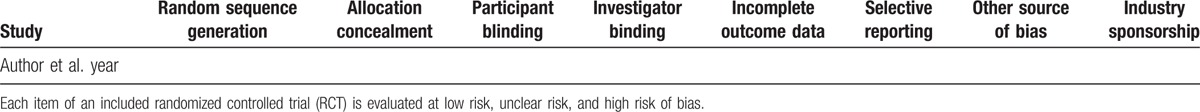
Risk of bias and sponsorship of included studies.

### 2.8 Data synthesis and statistical analysis

#### 2.8.1 Pairwise meta-analyses

We will perform pairwise meta-analysis using random-effects model firstly. Mean difference (MD) for continuous outcomes and odds ratio (OR) for dichotomous outcomes will be employed to estimate relative curative effects of the competing interventions, both with 95% confidence interval (CI). The statistical heterogeneity among studies will be assessed by the Cochran Q test and the *I*^2^ statistic. A *P* value of 0.05 or less for the Q test or an *I*^2^ greater than 50% indicates substantial study heterogeneity.^[23]^ We will use STATA statistical software, V.14 (Stata Corp, College Station, TX) to perform all analysis.

#### 2.8.2 Network meta-analyses

For indirect and mixed comparisons, we will conduct random-effects Bayesian NMA employing Markov chain Monte Carlo methods by WinBUGS version 1.4.3 which use informative prior distributions for all treatment effects as well as the between-study variance parameter.^[24]^ The results of NMA with effect sizes (MD or RR) and their credible intervals (CrI) will be summarized. The pooled estimates can be obtained by means of the Markov Chains Monte Carlo method. Four Markov chains can be run synchronously with various arbitrarily chosen initial values. We will estimate the relative ranking probability of each strategy and obtain the hierarchy of competing interventions using rankograms.^[25]^

### 2.9 Exploration of inconsistency

To check for inconsistency, the loop-specific approach will be performed on behalf of assessing the diversity between direct and indirect estimates for a particular comparison in the loop.^[26]^ We will employ the node-splitting method, excluding 1 direct comparison at a time and assessing the indirect treatment effect due to the excluded comparison. The design-by-treatment model will be conducted to check for the assumption of consistency.^[27]^ We will explore the possible source if important inconsistency is presenting. We will run network meta-regression analyses to account for differences by time since stroke, sex, dietary assessment method, baseline HAMD score, hemisphere stroke status, and depression class, if sufficient data will be available

### 2.10 Subgroup effects analysis and sensitively analysis

We will estimate subgroup effects, including participants baseline characteristics (eg, ethnic groups, age, and severity of depression) within individual trials and combining these data across studies. In particular, for pharmacotherapy trials, we will treat alternative dosing or duration schemes of the same drug as different nodes in the network, in order to investigate potential dose–response and duration–response associations. To examine the robustness of our results, we will restrict to RCTs with a low risk of bias for sequence generation during sensitively analysis, as well as blinding components of the Cochrane risk of bias tool.

## 3 Discussion

Our review will provide the most comprehensive synthesis for available interventions for PSD. To the best of our knowledge, this will be the 1st NMA that will include all available PSD interventions and pharmaceutical agents. The results will be of interest to a broad audience: neurologists, psychiatrist, practice guide-line developers, and policy-makers, because it could be recruited to give clinical recommendations for patients with PSD. Novel method for rating the confidence in the estimates will be employed with us which was recommended by the GRADE working group. On the other hand, several drawbacks should be noted in this study. We will anticipate some extent of clinical heterogeneity considering the possible sources that we described. Furthermore, the ability to explore heterogeneity may be limited in case of the small number of included studies.

## Acknowledgments

The authors thank Ian Charles Tobias for reviewing the manuscript.

## Supplementary Material

Supplemental Digital Content
