# Supplementary material for: Pharmacological and psychotherapeutic interventions for management of poststroke depression: A Bayesian network meta-analysis of randomized controlled trials
Source: Medicine (Baltimore). 2017 Feb 17;96(7):e6100. doi: 10.1097/MD.0000000000006100 (PMC5319512; doi:10.1097/MD.0000000000006100)
Supplement: Supplemental Digital Content [file medi-96-e6100-s001.docx]

Contents of Supplementary appendix

**Appendix: Search algorithms**

| Medline | |
| --- | --- |
| #1 | “randomized controlled trial” [Publication Type] |
| #2 | “controlled clinical trial” [Publication Type] |
| #3 | “randomized” [Title/Abstract] |
| #4 | “randomly” [Title/Abstract] |
| #5 | “trial” [Title] |
| #6 | “Randomized Controlled Trial as Topic” [MeSH] |
| #7 | (#1) OR (#2) OR (#3) OR (#4) OR (#5) OR (#6) |
| #8 | “PSD” [Title/Abstract] |
| #9 | “post-stroke depression” [Title/Abstract] |
| #10 | “post-stroke depressive”[Title/Abstract] |
| #11 | “depression after stroke”[Title/Abstract] |
| #12 | “depression in stroke patients”[Title/Abstract] |
| #13 | “depression after cerebral apoplexy”[Title/Abstract] |
| #14 | “depression after cerebrovascular accident”[Title/Abstract] |
| #15 | “depression after cerebrovascular disease”[Title/Abstract] |
| #16 | (#8) OR (#9) OR (#10) OR (#11) OR (#12) OR (#13) OR (#14) OR (#15) |
| #17 | “Therapeutics”[Mesh] OR “Antidepressive Agents” [Mesh] |
| #18 | “Serotonin Uptake Inhibitors” [Mesh] OR “Fluoxetine” [Mesh] OR “Sertraline” [Mesh] OR “Paroxetine” [Mesh] OR “Citalopram” [Mesh] OR “Fluvoxamine” [Mesh] OR “Escitalopram” [Title/Abstract] |
| #19 | “Antidepressive Agents, Tricyclic” [Mesh] OR “Nortriptyline” [Mesh] OR “Imipramine” [Mesh] OR “Clomipramine” [Mesh] OR “Amitriptyline” [Mesh] |
| #20 | “serotonin norepinephrine reuptake inhibitor” [Title/Abstract] OR “SNRI” [Title/Abstract] OR “Venlafaxine” [Mesh] OR “Duloxetine” [Mesh] OR “NRI” [Title/Abstract] OR “reboxetine” [Title/Abstract] |
| #21 | “Monoamine Oxidase Inhibitors” [Mesh] OR “Methylphenidate” [Mesh] OR “aniracetam” [Title/Abstract] OR “psychostimulant” [Title/Abstract] |
| #22 | “Drugs, Chinese Herbal” [Mesh] OR “Acupuncture Therapy” [Mesh] |
| #23 | “Psychotherapy” [Mesh] OR “Behavior Therapy” [Mesh] |
| #24 | “Transcranial Magnetic Stimulation” [Mesh] OR “Electroconvulsive Therapy” [Mesh] |
| #25 | “Mindfulness” [Mesh] OR “Music Therapy” [Mesh] OR “General Surgery” [Mesh] OR “Rehabilitation” [Mesh] OR “Social Support” [Mesh] OR “Education” [Mesh] OR “Family” [Mesh] OR “Nurses” [Mesh] |
| #26 | (#17) OR (#18) OR (#19) OR (#20) OR (#21) OR (#22) OR (#23) OR (#24) OR (#25) |
| #27 | (#7) AND (#16) AND (#26) |
| Total number # | |

| Embase | |
| --- | --- |
| #1 | ‘randomized controlled trial’/exp: ti,ab,kw |
| #2 | ‘randomized controlled trial (topic)’/exp: ti,ab,kw |
| #3 | random *: ti,ab,kw |
| #4 | #1 OR #2 OR #3 |
| #5 | ‘PSD’/exp: ti,ab,kw |
| #6 | ‘post-stroke depression’/exp: ti,ab,kw: ti,ab,kw |
| #7 | ‘post-stroke depressive’/exp: ti,ab,kw |
| #8 | ‘depression after cerebral apoplexy’/exp: ti,ab,kw |
| #9 | ‘depression after cerebrovascular accident’/exp: ti,ab,kw |
| #10 | ‘depression after cerebrovascular disease’/exp: ti,ab,kw |
| #11 | ‘depression in stroke patients’/exp: ti,ab,kw |
| #12 | ‘depression after stroke’ /exp: ti,ab,kw |
| #13 | #5 OR #6 OR #7 OR #8 OR #9 OR #10 OR #11 OR #12 OR |
| #14 | ‘therapeutics’ /exp: ti,ab,kw OR ‘antidepressive Agents’ /exp: ti,ab,kw |
| #15 | ‘tricyclic’ /exp: ti,ab,kw |
| #16 | ‘serotonin uptake inhibitors’ /exp: ti,ab,kw |
| #17 | ‘serotonin norepinephrine reuptake inhibitor’ /exp: ti,ab,kw |
| #18 | ‘monoamine oxidase inhibitors’ /exp: ti,ab,kw |
| #19 | ‘fluoxetine’ /exp: ti,ab,kw OR ‘sertraline’ /exp: ti,ab,kw OR ‘paroxetine’ /exp: ti,ab,kw OR ‘citalopram’ /exp: ti,ab,kw |
| #20 | ‘Chinese Herbal’ /exp: ti,ab,kw OR ‘acupuncture’ /exp: ti,ab,kw |
| #21 | ‘psychotherapy’ /exp: ti,ab,kw OR ‘behavior therapy’ /exp:ti,ab,kw OR ‘transcranial magnetic stimulation’ /exp: ti,ab,kw OR ‘electroconvulsive therapy’ /exp: ti,ab,kw |
| #22 | ‘mindfulness’ /exp: ti,ab,kw OR ‘music therapy’ /exp: ti,ab,kw OR ‘surgery’ /exp: ti,ab,kw OR ‘rehabilitation’ /exp: ti,ab,kw OR ‘education’ /exp: ti,ab,kw |
| #23 | (#14) OR (#15) OR (#16) OR (#17) OR (#18) OR (#19) OR (#20) OR (#21) OR (#22) |
| #24 | (#4) AND (#13) AND (#23) |
| Total number # | |

| Cochrane Library Central | |
| --- | --- |
| #1 | PSD: ti, ab, kw OR post- stroke depression: ti, ab, kw in Trials (Word variations have been searched) |
| #2 | depression after cerebrovascular disease: ti, ab, kw OR depression after cerebral apoplexy: ti, ab, kw in Trials (Word variations have been searched) |
| #3 | depression after stroke: ti, ab, kw in Trials (Word variations have been searched) |
| #4 | depression in stroke patients : ti, ab, kw in Trials (Word variations have been searched) |
| #5 | (#1) OR (#2) OR (#3) OR (#4) |
| #6 | therapeutics: ti, ab, kw OR antidepressive: ti, ab, kw OR serotonin reuptake inhibitor: ti, ab, kw OR tricyclic: ti, ab, kw OR monoamine oxidase inhibitor: ti, ab, kw in Trials (Word variations have been searched) |
| #7 | fluoxetine: ti, ab, kw OR sertraline: ti, ab, kw OR paroxetine: ti, ab, kw OR citalopram: ti, ab, kw OR reboxetine: ti, ab, kw in Trials (Word variations have been searched) |
| #8 | trazodone: ti, ab, kw OR nortriptyline: ti, ab, kw OR escitalopram: ti, ab, kw OR psychostimulant: ti, ab, kw in Trials (Word variations have been searched) |
| #9 | Chinese herbal medicine: ti, ab, kw OR acupuncture: ti, ab, kw OR behavior therapy: ti, ab, kw OR psychotherapy: ti, ab, kw in Trials (Word variations have been searched) |
| #10 | transcranial magnetic stimulation: ti, ab, kw OR surgery: ti, ab, kw OR electroconvulsive therapy: ti, ab, kw OR mindfulness: ti, ab, kw OR music therapy: ti, ab, kw in Trials (Word variations have been searched) |
| #11 | nurse: ti, ab, kw OR care: ti, ab, kw OR support: ti, ab, kw OR family: ti, ab, kw OR education: ti, ab, kw in Trials (Word variations have been searched) |
| #12 | (#6) OR (#7) OR (#8) OR (#9) OR (#10) OR (#11) |
| #13 | (#5) AND (#12) |
| Total number # | |
